# Inter-population variations in concentrations, determinants of and correlations between 2,2',4,4',5,5'-hexachlorobiphenyl (CB-153) and 1,1-dichloro-2,2-bis (*p*-chlorophenyl)-ethylene (p,p'-DDE): a cross-sectional study of 3161 men and women from Inuit and European populations

**DOI:** 10.1186/1476-069X-4-27

**Published:** 2005-11-11

**Authors:** Bo AG Jönsson, Lars Rylander, Christian Lindh, Anna Rignell-Hydbom, Aleksander Giwercman, Gunnar Toft, Henning S Pedersen, Jan K Ludwicki, Katarzyna Góralczyk, Valentyna Zvyezday, Marcello Spanò, Davide Bizzaro, Eva C Bonefeld-Jörgensen, Gian Carlo Manicardi, Jens Peter Bonde, Lars Hagmar, Inuendo

**Affiliations:** 1 Department of Occupational and Environmental Medicine, Lund University Hospital, SE-221 85 Lund, Sweden; 2 Scanian Fertility Centre, Malmö University Hospital, Malmö, SE-205 02 Sweden; 3 Department of Occupational Medicine, Aarhus University Hospital, Nørrebrogade 44, build. 2C, DK-8000 Aarhus C, Denmark; 4 Centre for Arctic Environmental Medicine, Postbox 570DK-3900 Nuuk, Greenland, Denmark; 5 Department of Environmental Toxicology, National Institute of Hygiene, Warsaw, Chocimska 24, P-00-791 Warsaw, Poland; 6 Laboratory of Human Reproduction, Kharkiv State Medical University, Klochkovskaya Street 156-A, room 14, 61145 Kharkiv, Ukraine; 7 Section of Toxicology and Biomedical Sciences, BIOTEC-MED, ENEA CR Casaccia, Via Anguillarese 301, 00060 Rome, Italy; 8 Istituto di Biologia e Genetica, Università Politecnica delle Marche, Via Brecce Bianche, I-60131 Ancona, Italy; 9 Institute of Public Health, Department of Environmental and Occupational Medicine, University of Aarhus, University of Aarhus, Vennelyst Boulevard 6, DK-8000 Aarhus C, Denmark; 10 Laboratorio di Genetica, Dip. di Science Agrarie, Università di Modena e Reggio Emilia, Viele Kennedy 17 – Reggio Emilia I-41100 Modena, Italy

## Abstract

**Background:**

The study is part of a collaborative project (Inuendo), aiming to assess the impact of dietary persistent organochlorine pollutants (POPs) on human fertility. The aims with the present study are to analyze inter-population variations in serum concentrations of 2,2',4,4',5,5'-hexachlorobiphenyl (CB-153) and 1,1-dichloro-2,2-bis (*p*-chlorophenyl)-ethylene (p,p'-DDE), to assess inter-population variations in biomarker correlations, and to evaluate the relative impact of different determinants for the inter-individual variations in POP-biomarkers.

**Method:**

In study populations of 3161 adults, comprising Greenlandic Inuits, Swedish fishermen and their wives, and inhabitants from Warsaw, Poland and Kharkiv, Ukraine, serum concentrations of CB-153 and p,p'-DDE, were analysed by gas chromatography-mass spectrometry.

**Results:**

The median serum concentrations of CB-153 were for male and female Inuits 200 and 110, for Swedish fishermen 190 and their wives 84, for Kharkiv men and women 44 and 27, and for Warsaw men and women 17 and 11 ng/g lipids, respectively. The median serum concentrations of p,p'-DDE were for Kharkiv men and women 930 and 650, for male and female Inuits 560 and 300, for Warsaw men and women 530 and 380, and for Swedish fishermen 240 and their wives 140 ng/g lipids, respectively. The correlation coefficients between CB-153 and p,p'-DDE varied between 0.19 and 0.92, with the highest correlation among Inuits and the lowest among men from Warsaw. Men had averagely higher serum concentrations of CB-153 and p,p'-DDE, and there were positive associations between age and the POP-biomarkers, whereas the associations with BMI and smoking were inconsistent. Dietary seafood was of importance only in the Inuit and Swedish populations.

**Conclusion:**

CB-153 concentrations were much higher in Inuits and Swedish fishermen's populations than in the populations from Eastern Europe, whereas the pattern was different for p,p'-DDE showing highest concentrations in the Kharkiv population. The correlations between the POP-biomarkers varied considerably between the populations, underlining that exposure sources differ and that the choice of representative biomarkers of overall POP exposure has to be based on an analysis of the specific exposure situation for each population. Age and gender were consistent determinants of serum POPs; seafood was of importance only in the Inuit and Swedish populations.

## Introduction

The use of persistent organochlorine pollutants (POP), such as polychlorinated biphenyls (PCB), polychlorinated dibenzo-p-dioxins, polychlorinated dibenzofurans and pesticides such as dichlorodiphenyl trichloroethane (DDT) has been restricted and bans implemented in many countries during the 1970's and 1980's, but e.g. DDT is still used in some areas for malaria vector control. These ubiquitous lipophilic POPs are highly resistant to both abiotic and biotic degradation, and are transported long distances by air and water streams.

Some POPs can disrupt multiple endocrine pathways and induce a wide range of toxic responses [1]. A variety of studies have demonstrated their estrogenic, anti-estrogenic, and androgen competing properties [2-4]. There is also concern that POPs may adversely affect male fertility [5].

The present study is a part of a collaborative research project funded by EU (Inuendo; ) which aims to identify and characterize the impact of dietary POP on human fertility in an epidemiological setting [6]. The scope is to contribute scientific knowledge as basis for appropriate assessment and management of risks related to exposure to POP. Study populations have been established in three European countries; Sweden, Poland, and Ukraine together with a population of Inuits from Greenland. The choice of study populations was motivated by that in the Arctic, the combination of environmental conditions and biomagnification in the aquatic food chains result in accumulation of POP in local food at concentrations which are often in excess of contaminants in the mid-latitudes where these contaminants originate [7]. Also Swedish fishermen's families at the east coast at the Baltic Sea, with a high consumption of herring and salmon, constitute a highly exposed group [8,9]. However, in both these populations there are large inter-individual exposure contrasts. Both among Inuits and Swedish fishermen the serum concentrations of e.g. 2,2',4,4',5,5'-hexachlorobiphenyl (CB-153) and 1,1-dichloro-2,2-bis (*p*-chlorophenyl)-ethylene (p,p'-DDE) were in the same order of magnitude [7-9]. Much less is known about exposure levels for the general populations in Central and Eastern Europe [10]. However, previous data support that the p,p'-DDE concentrations was one order of magnitude higher than the CB-153 concentrations [11].

We have chosen to use CB-153 as a biomarker for POP exposure, because it correlated very well (r = 0.9) with both total PCB concentration in plasma and serum from Swedish subjects and Inuits from Greenland [12-15]. Another relevant biomarker is the anti-androgenic compound p,p'-DDE, the major metabolite of the insecticide DDT with a long biological half-live. Both compounds occur in relatively high concentrations in serum and they are feasible biomarkers in that sense that a large number of analyses can be performed with good precision and accuracy and to a reasonable cost.

The aims with this publication are to a) analyze inter-population variations in CB-153 and p,p'-DDE exposure concentrations and b) assess inter-population variations in biomarker correlations, c) and finally to assess the relative impact of different determinants (gender, age, residence area, breast feeding, smoking, body mass index [BMI], gestational length, and dietary intake of seafood) for the inter-individual variations in serum concentrations of CB-153 and p,p'-DDE.

## Subjects and methods

### Subjects and data collection

The aim was to recruit 600 pregnant women and their male spouses in Greenland, Warsaw, and Kharkiv, respectively, and 200 Swedish fishermen and 600 Swedish fishermen's wives. A general criterion for eligibility was that the participants had to be born in the country of study and to be at least 18 years of age.

In *Greenland *pregnant Inuit women from 15 municipalities and 4 settlements from all regions within the country were contacted and informed about the project. In total 893 pregnant women were listed at the local midwives, but 235 did not fulfill the inclusion criteria, 32 could not be contacted and 38 did not want to participate. Blood samples were drawn from the pregnant women and their spouses (all were Inuits), and interviews were made with both partners from June 2002 to December 2003. Of the 598 women included in the time to pregnancy (TTP) study (the results will be published elsewhere), 62 were living alone, 36 men could not be contacted, 34 men did not want to be interviewed, and 5 men did not want to give a blood sample. Moreover, for unknown reasons, blood samples were lost for 16 women and 22 men. Thus, in total, blood samples and interview data were available for 439 men (82 % participation rate) and 572 women (87 % participation rate) (Tables 1 and 2).

**Table 1:** Serum concentrations of CB-153 and p,p'-DDE (ng/g lipid) and potential confounders among the men.^a^

	Inuit men (n = 439)		Swedish fishermen (n = 189)		Warsaw men (n = 257)		Kharkiv men (n = 287)	
	
	M^b^	5–95 %	M	5–95 %	M	5–95 %	M	5–95 %
CB-153	200	50–920	190	62–640	17	<LOD^c^-38	44	5.5–130
p,p'-DDE	560	90–2200	240	80–890	530	220–1000	930	390–2400
Age (years)	30	21–44	48	32–62	30	25–37	25	20–39
BMI (kg/m^2^)	25.6	20.4–33.1	26.4	22.0–32.6	25.4	20.4–31.7	23.9	19.7–29.5
Smoking								
Never^d^	16		38		52		25	
Ever^d^	18		62		48		75	
Cig/day	8.0	0–20	11	0–25	0	0–20	7.5	0–20
Seafood (meals/week)	1.5	0.1–5.0	n.a^e^		1.0	0–3.0	4.0	2.0–5.0

a) Except area of residence

b) Median

c) Below detection level

d) %

e) n.a. = not analyzed because data was not available

**Table 2:** Serum concentrations of CB-153 and p,p'-DDE (ng/g lipid) and potential confounders among the women.^a^

	Inuit women (n = 572)		Swedish women (n = 544)		Warsaw women (n = 261)		Kharkiv women (n = 612)	
	
	M^b^	5–95 %	M	5–95 %	M	5–95 %	M	5–95 %
CB-153	110	21–530	84	30–220	11	<LOD^c^-27	27	8.1–69
p,p'-DDE	300	49–1300	140	50–530	380	140–880	650	270–1700
Age (years)	27	19–38	50	37–57	29	25–34	24	18–34
BMI (kg/m^2^)	23.8	18.9–32.8	24.8	20.3–32.9	21	18.1–27.3	24	17.6–27.7
Smoking			n.a.^e^					
Never^d^	16				81		82	
Ever^d^	84				19		18	
Cig/day	6	0–15			0	0–10	0	0–10
Seafood (meals/week)	1.2	0–5.0	0.4	0–1.3	1.0	0–3.0	0	0–3.0
Gestational length (weeks)	24	9–38	n.a.		33	9–38	22	7–41
Primipara^d^	32		n.a.		93		80	
Total lactation time (months)	n.a.		12	0–40	n.a.		n.a.	

a) Except area of residence

b) Median

c) Below detection level

d) %

e) n.a. = not analyzed because data was not available

Altogether 690 pregnant women and their spouses who either visited the obstetric out-patient clinic of the Gynecological and Obstetric Hospital of the *Warsaw *Medical University, Poland, or physicians at a collaborating hospital in the same city were informed about the project and asked to participate. Of those, 261 (38 % participation rate) women and 257 (37 % participation rate) men donated a blood sample and were interviewed from September 2002 to March 2003 (Tables 1 and 2). The population was urban.

Altogether 2478 pregnant women and their spouses who visited one of eight antenatal clinics or three maternity hospitals in *Kharkiv*, Ukraine, were informed about the project and asked to participate. Of those, 612 (25 % participation rate) women and 287 (12 % participation rate) men donated a blood sample and were interviewed from April 2003 to December 2004. The population had a mixed urban and rural background.

In contrast to the other three participating populations, the recruitment process in *Sweden *was not focused on ongoing pregnancies. Instead blood samples were drawn from 189 professional fishermen from the Swedish east and west coasts cohorts that participated in a semen study between March 2001 and November 2001, and March 2002 and September 2002 (Tables 1 and 2). Initially 2614 Swedish fishermen had been informed about the semen study and 266 (10 % participation rate) gave their written informed consent to participate, but 71 subjects had to be excluded during the sampling period due to logistical reasons, changes of mind, sickness or recent vasectomy during the field study period [16]. Altogether 1439 fishermen's wives, born 1945 or later, were contacted for a TTP study. Blood samples were drawn from, and interviews were performed on 544 (38 % participation rate) women between December 2002 and March 2004. Eighty-seven spouses to the fishermen that participated in the semen study were eligible and 68 of them participated.

The study was approved by the local ethical committees representing all participating populations and all subjects signed an informed consent.

### Interview data

Information on lifestyle, medical and reproductive history was collected through interviews. In the present study we used information on age, gestational length, height and weight (for calculation of BMI), smoking habits, and intake of seafood. For the Inuits seafood comprised sea mammals as well as fish, whereas for the other populations it only refers to fish. For the fishermen's wives from Sweden information on life-long total length of breast-feeding was obtained and for the other women whether it was the first time they were given birth (primipara). For the populations from Sweden and Greenland we also used information regarding area of living. Information on current smoking was not obtained for the women from Sweden and information on current intake of seafood was not obtained for the Swedish fishermen.

### Collection of blood samples

Blood samples were drawn from a cubital vein into 10 ml vacuum tubes for serum collection without additives (Becton Dickinson, Maylan, France). After cooling to room temperature the tubes were centrifuged at 4000 *g *for 15 min. Serum was transferred with ethanol rinsed Pasteur pipettes to ethanol rinsed brown glass bottles (Termometerfabriken, Gothenburgh, Sweden). A piece of aluminum foil was placed on top of the bottles which were then sealed. Sera were stored at -20°C until shipment, but it was accepted to keep it in refrigerator for up to four days.

### Determination of CB-153 and p,p'-DDE in serum

The sera were transported on dry ice to the Department of Occupational and Environmental Medicine in Lund, Sweden, where all analysis of CB-153 and p,p'-DDE were performed. The CB-153 and p,p'-DDE were extracted from serum by solid phase extraction using on-column degradation of the lipids and analysis by gas chromatography mass spectrometry as previously described [16]. The relative standard deviations, calculated from samples analyzed in duplicate at different days, was 18% at 0.1 ng/mL (n = 990), 10 % at 0.5 ng/mL (n = 990) and 10 % at 2 ng/mL (n = 990) for CB-153 and 11 % at 1 ng/mL (n = 1058), 8 % at 3 ng/mL (n = 1058) and 7 % at 8 ng/mL (n = 1058) for p,p'-DDE. The detection limits were 0.05 ng/mL for CB-153 and 0.1 ng/mL for p,p'-DDE. The analyses of CB-153 and p,p'-DDE were part of the Round Robin inter-comparison program (Prof. Hans Drexler, Institute and Out-Patient Clinic for Occupational, Social and Environmental Medicine, University of Erlangen-Nuremberg, Germany) with analysis results within the tolerance limits. An internal control at 0.4 ng/mL for CB-153 and 0.6 ng/mL for p,p'-DDE was included in all analyzed series. The samples were analyzed in duplicate and the samples were re-analyzed a third time if the difference between the two determinations were higher than 30 %. However, at concentrations below 0.2 ng/mL an absolute deviation of 0.1 ng/mL between the duplicate samples was allowed. After the third analysis the two determinations fulfilling the above criteria was chosen. If none of the determinations fulfilled the criteria the sample were analyzed a fourth time.

### Determination of lipids by enzymatic methods

Serum concentrations of triglycerides and cholesterol were determined by enzymatic methods using reagents from Roche Diagnostics (Mannheim, Germany). The inter-assay CVs for cholesterol and triglyceride determinations were 1.5–2.0 %. The average molecular weights of triglycerides were assumed to be 807. For cholesterol we used an average molecular weight of 571, assuming that the proportion of free and esterified cholesterol in plasma was 1:2. Based on a paper by Rylander et al., the total lipid concentration in serum (g/L) was calculated by the following equations [17]:

Men: Total = 0.96 + 1.28*(triglycerides + cholesterol)

Women: Total = 1.13 + 1.31*(triglycerides + cholesterol).

### Statistical analysis

Separate analyses for each population were performed for men and women. If the concentration was less than the limit of detection, the concentration was set to half of the detection limit based on the fresh weight concentrations. For CB-153 there were 122 (men: 37; women: 85) and for p,p'-DDE there were 13 (men: 3; women: 10) values below the limits of detection. The impact of potential determinants for inter-individual variations in CB-153 and p,p'-DDE serum concentrations were assessed by linear regression models. We also evaluated whether log transformations of the POP variables better fulfilled the model assumptions, which were checked by analysis of residuals according to the methods suggested by Altman [18]. The determinants evaluated were age (continuous), BMI (continuous), smoking (categorical: ever/never; continuous: cigarettes/day), and number of meals of seafood per week (not for Swedish fishermen). The impact of area of residence was assessed for the Inuits (three categories: Living in the capital Nuuk [presumed low exposure], living on the remaining west coast [presumed medium exposure] and living in the north or east coast regions [presumed high exposure]) and the Swedish study population (two categories: west [presumed low exposure] and east [presumed high exposure] coasts). In addition, we also evaluated the impact of gestational length at blood sampling and parity (primipara: yes/no) for women from Greenland, Warsaw and Kharkiv. Among Swedish fishermen's wives the impact of life-long total length of breast-feeding was assessed (four categories: 0–6, >6–12, >12–24 and >24 months).

The objective of the multivariate model building was to evaluate the impact of each of the determinants. In the first step we evaluated the associations for one determinant at a time with the CB-153 and p,p'-DDE concentrations in serum, respectively. In the second step we kept the determinant with the lowest p-value (if ≤ 0.15) and tentatively included all other determinants, one at a time. In the third step we kept the two determinants with the lowest p-values (if both ≤ 0.15) and tentatively included the remaining determinants, one at a time. This procedure was continued as long as the p-values for the all included determinants were ≤0.15. For the populations from Greenland and Sweden (only among the women) we also evaluated whether area of living modified the effect of seafood by including an interaction term in the models. The explained variances (adjusted r^2 ^obtained from SPSS™), after successive addition of determinants to the final multivariate model, are given.

The inter-population variations in CB-153 and p,p'-DDE serum concentrations were also assessed by linear regression models. In these models age was considered as a potential confounder. The age distribution among the women from Sweden differed so much from the other age distributions (with almost no overlap) and we did therefore not make age-adjusted comparisons for the women from Sweden.

The correlations between serum concentrations of CB-153 and p,p'-DDE for the different populations were evaluated by Spearman's correlation coefficients.

## Results

### Inter-population variations in serum concentrations of CB-153 and p,p-DDE

In total, the material included lipid adjusted serum concentrations of CB-153 and p,p'-DDE for 1172 men and 1989 women. In Tables 1 and 2, the concentrations and the distributions of CB-153 and p,p'-DDE in the different populations are given. In Table 3 the serum concentrations of the POP markers are given by area of residence within Greenland and Sweden.

**Table 3:** Serum concentrations of CB-153 and p,p'-DDE (ng/g lipid) with respect to area of residence. Data for men and women from Greenland and Sweden, respectively.

	Men			Women		
	
	n	M^a^	5–95 %	n	M	5–95 %
**CB-153**						
**Greenland**						
Nuuk	122	130	38–680	159	79	11–328
West^b^	286	220	55–810	377	120	23–490
East/North^c^	29	600	110–1900	33	490	77–1400

**Sweden**						
West coast	97	170	55–370	360	79	32–160
East coast	92	210	63–800	184	97	27–260

**p,p-DDE**						

**Greenland**						
Nuuk	122	340	47–1600	159	240	15–780
West^b^	286	610	150–2200	377	310	59–1100
East/North^c^	29	1900	310–5000	33	1000	230–2400

**Sweden**						
West coast	97	200	62–620	97	200	62–620
East coast	92	290	110–1200	92	290	110–1200

a) median

b) Aasiaat, Ilulissat, Kangaatsiaq, Maniitsoq, Nanortalik, Narsaq, Paamiut, Qaqortoq, Qasigiannguit, Qeqertarsuaq, Saattut, Sisimiut, Ukkusissat and Uummannaq

c) Qaanaaq, Tasiilaq, Kulusuk and Kuummiut

For the men the mean serum concentrations of CB-153 was in the following order: Inuits (highest), Swedish fishermen, Warsaw and Kharkiv (lowest) (Table 1). The crude mean difference between Inuits and Swedish fishermen was 78 ng/g lipid (95 % CI 19, 137, p < 0.001), and this difference became even more evident after age adjustment (mean difference 297 ng/g lipid; 95 % CI 220, 375, p < 0.001), indicating that the Inuits had reached high serum concentrations of CB-153 already in their twenties. The fishermen from Sweden had higher concentrations as compared with the men from Kharkiv (age adjusted mean difference: 43, 95 % CI 3.5, 82), which in turn had higher concentrations as compared with the men from Warsaw (age adjusted mean difference 43 ng/g lipid, 95 % CI 33, 53).

The pattern among the males with respect to population was quite different for p,p'-DDE (Table 1). The men from Kharkiv had significantly higher concentrations than the Inuits (age adjusted mean difference 457 ng/g lipid, 95 % CI 309, 605), which in turn had significantly higher concentrations than the men from Warsaw (age adjusted mean difference 213 ng/g lipid, 95 % CI 96, 329), and finally the Swedish fishermen had significantly lower concentrations than the men from Warsaw (age adjusted mean difference 426 ng/g lipid, 95 % CI 339, 514).

The Inuit women had significantly higher serum concentrations of CB-153 as compared with the women from Kharkiv (age-adjusted mean difference 133 ng/g lipid, 95 % CI 116, 150), whom in turn had higher concentrations than the women from Warsaw (age-adjusted mean difference 24 ng/g lipid, 95 % CI 20, 28).

The women from Kharkiv had significantly higher age-adjusted serum concentrations of p,p'-DDE as compared with the Inuit women and the women from Warsaw (all p-values<0.001). There was, however, no age-adjusted difference between the Inuit women and the women from Warsaw (p = 0.17).

### Inter-population variations in biomarker correlations

The correlation coefficients (r_s_) between serum concentrations of CB-153 and p,p'-DDE in the different populations varied between 0.19 and 0.92, with the highest correlation among Inuits and the lowest correlation among men from Poland (Table 4).

**Table 4:** Correlations between serum concentrations of CB-153 and p,p'-DDE.^a^

	Inuits	Swedish fishermen's populations	Warsaw	Kharkiv	Total
Men, r_s_^b^	0.93	0.79	0.19	0.42	0.17
Women, r_s_^b^	0.92	0.76	0.54	0.49	0.03

a) For men and women for each of the populations, and for the total study group

b) Spearman's correlation coefficient

### Impact of determinants for inter-individual variations in serum POP concentrations

There was a positive association between *age *and serum concentrations of CB-153 in all male populations, although the impact of an increase in age of one year meant more in Inuits (β = 16 ng/g lipid) and Swedish fishermen (β = 9.0) as compared with men from Warsaw (β = 1.2) and Kharkiv (β = 2.0) (Tables 5, 6, 7, 8). The quantitative effect of age can be exemplified by that the serum concentrations of CB-153 increased in the Inuits by 16 ng/g lipids for each extra year of age, whereas the similar age-related increase was only 1.2 ng/g lipids among the men from Warsaw. Age was also positively associated (p < 0.001) with p,p'-DDE serum concentrations in the Inuit and Swedish populations (Tables 5 and 6). In concordance with the results for men, also among the women age was positively associated with POP concentrations in all populations (all p-values = 0.01, Tables 5, 6, 7, 8).

**Table 5:** Linear multiple regressions for determinants for serum concentrations of CB-153 (ng/g lipid) and p,p'-DDE (ng/g lipid) among Inuit men and women.^a^

	Inuit men		Inuit women	
	
	CB-153	p,p'-DDE	CB-153	p,p'-DDE
Age, years	16 (12, 21)	31 (20, 42)	7.7 (5.2, 10)	15 (9.0, 21)
BMI, kg/m^2^	-^b^	-	-2.9 (-6.1, 0.2)	-^b^
Current smoking				
No	-	-	Ref	Ref
Yes	-	-	36 (-1.5, 75)	101 (13, 189)
Cig/day	-	-	-	-
Seafood, meals/week	53 (33, 73)	130 (80, 179)	18 (8.8, 28)	58 (36, 80)
Area				
Nuuk	Ref	Ref	Ref	Ref
West	87 (13, 162)	309 (127, 491)	46 (14, 78)	94 (20, 168)
East/North	597 (454, 740)	1271 (922, 1620)	399 (335, 463)	773 (626, 921)
Primipara				
No			Ref	Ref
Yes			26 (-7.5, 60)	60 (-18, 138)
Gestational length (weeks)			-	-
Explained variance^c^(%)	12 (area) 24 (+age) 27 (+seafood)	12 (area) 18 (+age) 22 (+seafood)	21 (age) 26 (+area) 28 (+cig/day)	16 (area) 21 (seafood) 24 (+age) 25 (+smoking) 25 (+parity)

a) The β-coefficients (with 95 % CI within brackets) are shown.

b) The variable did not fulfil the criteria to be included in the final model.

c) Cumulative explained variance after successive addition of determinant variables to the final multivariate model. Thus, the total explained variance is given on the lowest line.

**Table 6:** Linear multiple regressions for determinants for serum concentrations of CB-153 (ng/g lipid) and p,p'-DDE (ng/g lipid) among Swedish fishermen and their wives.^a^.

	Swedish fishermen		Fishermens' wives			
	
	CB-153	p,p'-DDE	CB-153		p,p'-DDE	
			
			East coast	West coast	East coast	West coast
Age, years	9.0 (6.5, 12)	10 (6.1, 14)	6.3 (4.7, 8.0)	2.9 (2.2, 3.6)	18 (13, 22)	5.4 (3.7, 7.2)
BMI, kg/m^2^	-^b^	13 (0.3, 25)	-	-	6.2 (-0.7, 13)	3.0 (-0.1, 6.1)
Current smoking			n.a.^c^	n.a.	n.a.	n.a.
No	-	-				
Yes	-	-				
Cig/day	-2.9 (-5.1, -0.6)	-				
Seafood, meals/week	n.a.		49 (23, 75)	17 (6.4, 28)	112 (35, 189)	-
Area						
West coast	Ref	Ref				
East coast	96 (48, 143)	160 (80, 241)				
Lactation (months)					-	-
0–6			Ref	Ref		
>6–12			3.7 (-23, 30)	1.7 (-11, 14)		
>12–24			10 (-16, 37)	-16 (-29, -5.0)		
>24			-35 (-64, -5)	-33 (-46, -19)		
Explained variance^d^(%)	21 (age) 26 (+area) 28 (+cig/day)	12 (age) 20 (+area) 21 (+BMI)	28 (age) 33 (+seafood) 36 (+lactation)	20 (age) 25 (+lactation) 27 (+seafood)	21 (age) 26 (+area) 28 (+cig/day)	16 (area) 21 (seafood) 24 (+age) 25 (+smoking) 25 (+parity)

a) The β-coefficients (with 95 % CI within brackets) are shown.

b) The variable did not fulfil the criteria to be included in the final model.

c) n.a = not analyzed because data was not available.

d) Cumulative explained variance after successive addition of determinant variables to the final multivariate model. Thus, the total explained variance is given on the lowest line.

**Table 7:** Linear multiple regressions for determinants for serum concentrationsof CB-153 (ng/g lipid) and p,p'-DDE (ng/g lipid) among Warsaw men and women.^a^

	Warsaw men		Warsaw women	
	
	CB-153	p,p'-DDE	CB-153	p,p'-DDE
Age, years	1.2 (0.8, 1.6)	-	1.3 (0.9, 1.6)	21 (9.6, 32)
BMI, kg/m^2^	-0.6 (-1.1, -0.1)	-10 (-21, 0.8)	-	-
Current smoking	-^a^	-		
No	-	-	-	Ref
Yes			-	-90 (-176,-3.9)
Cig/day	-	-	-0.2 (-0.4, 0.05)	-
Seafood, meals/week	-	-	1.1 (0.1, 2.1)	-
Primipara				-
No			Ref	
Yes			7.4 (2.8, 12)	
Gestational length (weeks)			-	-
Explained variance^c^(%)	12 (area) 24 (+age) 27 (+seafood)	12 (area) 18 (+age) 22 (+seafood)	14 (age) 19 (+parity) 19 (+seafood) 19 (+cig/day)	6 (age) 7 (+smoking)

a) The β-coefficients (with 95 % CI within brackets) are shown.

b) The variable did not fulfil the criteria to be included in the final model.

c) Cumulative explained variance after successive addition of determinant variables to the final multivariate model. Thus, the total explained variance is given on the lowest line.

**Table 8:** Linear multiple regressions for determinants for serum concentrations of CB-153 (ng/g lipid) and p,p'-DDE (ng/g lipid) among Kharkiv men and women. ^a^.

	Kharkiv men		Kharkiv women	
	
	CB-153	p,p'-DDE	CB-153	p,p'-DDE
Age, years	2.0 (0.4, 3.5)	-	1.4 (0.8, 2.0)	19 (7.6, 31)
BMI, kg/m^2^	-^b^	-	-0.8 (-1.6, -0.1)	-
Current smoking				
No	-	Ref	-	-
Yes	-	-200 (-458, 57)	-	-
Cig/day	-	-	-	-
Seafood, meals/week	-	-	-	-
Primipara				
No			Ref	Ref
Yes			5.5 (-1.5, 12)	135 (-2.7, 273)
Gestational length (weeks)			-0.3 (-0.5, -0.1)	-7.9 (-12, -4.0)
Explained variance^c^(%)	2 (age)	1 (cig/day)	3 (age) 4 (+gestational length) 5 (+BMI) 5 (+parity)	2 (gestational length) 4 (+age) 4 (+parity)

a) The β-coefficients (with 95 % CI within brackets) are shown.

b) The variable did not fulfil the criteria to be included in the final model.

c) Cumulative explained variance after successive addition of determinant variables to the final multivariate model. Thus, the total explained variance is given on the lowest line.

Regarding *BMI *and *smoking *the pattern was very inconsistent, e.g. the effect of BMI on serum concentrations of p,p'-DDE went in different directions among the Swedish fishermen and the men from Warsaw (Tables 6 and 7). Also among the women the patterns for BMI and smoking with respect to POP concentrations were very inconsistent (Tables 5, 6, 7, 8).

In both Inuit men and women and among the Swedish fishermen the *area of residence *was of highly significant importance (all p-values<0.001) for the POP concentrations (Tables 5 and 6).

Among Inuit men the number of *seafood *meals per week was positively associated with the serum concentrations of CB-153 (p < 0.001) as well as with the serum concentrations of p,p'-DDE (p < 0.001) (Table 5). Similar significant associations (p < 0.001) were seen among the Inuit women, and also for serum concentrations of CB-153 among the women from Warsaw (p = 0.03) (Table 7). Among the Swedish fishermen's wives there was a significant interaction (p < 0.001) between intake of seafood and area of residence (Table 6). The intake of seafood had a major impact on fishermen's wives from the Swedish east coast, whereas the association was less obvious among the west coast women. There was no such interaction between intake of seafood and area of residence among the Inuit women.

The women who were *giving birth to their first child *had in general higher concentrations of POP concentrations, although most p-values were above 0.05 (Tables 5, 7, 8). In the Swedish population, the fishermen's wives with the longest total life-time length of *breast-feeding *had lower concentrations of POP, with the exception of p,p'-DDE concentrations among the east coast women (Table 6). A significant negative association between *gestational length *at blood sampling and POP in serum was observed only for women from Kharkiv, corresponding to a weakly decrease of CB-153 with 0.3 ng/g and of p,p'-DDE with 7.9 ng/g (Table 8).

The *explained variances *in the final regression models among the men from the different populations varied between 1 and 28 %, and for the different female populations it varied between 4 and 36 % (Tables 5, 6, 7, 8). By log transformation of the POP variables the model assumptions were somewhat better fulfilled in the Swedish population. However, in the three other populations no such improvements were achieved by log transformation of the POP variables, and we do therefore present the results for the untransformed variables for all four populations.

## Discussion

As could be expected serum concentrations of CB-153 were much higher in the Inuit and Swedish fishermen's populations as compared with the populations from Eastern Europe, whereas the exposure pattern was different for p,p'-DDE showing highest concentrations in the Kharkiv population and relatively low concentrations in the Swedish fishermen's populations. It has to be borne in mind that the participants from Sweden were not recruited from the general Swedish population but from a fishermen's population with averagely higher POP exposures. This was done in order to ensure a sufficient exposure contrast within the total study population. Moreover, the age distribution in the Swedish fishermen's populations differed a lot from the age distributions among the populations from the other countries. Thus, direct comparisons between the Swedish and the other populations must therefore be interpreted with caution.

The exposure pattern in the Kharkiv population with relatively high p,p'-DDE concentrations and relatively low CB-153 concentrations are in accordance with a previous analysis of maternal milk from Ukraine, donated 1993–1994, in which the median p,p'-DDE concentration (2457 ng/g lipid) was one order of magnitude higher than the median CB-153 concentration (149 ng/g lipid) [11]. The most reasonable explanation for this is that the use of the insecticide DDT has ceased relatively late in the Ukraine.

A noteworthy result of the present study was that the degree of correlation between the two POP biomarkers, CB-153 and p,p'-DDE, varied considerably between the different populations. The extremely high correlation coefficients (r_s _0.92 and 0.93, respectively; Table 4) for the Inuits and the high correlation coefficients for the Swedish fishermen's populations (r_s _0.79 and 0.76, respectively) support a common exposure source for both contaminants. This was not that obvious for the populations from Warsaw and Kharkiv, for which there were no previous data concerning the inter-correlation between CB-153 and p,p'-DDE. These large differences in correlation underline that the choice of representative biomarkers of overall POP exposure has to be based on an analysis of the specific exposure situation for each population. It might seem paradoxical that the correlation coefficients between the two exposure biomarkers became considerably lower when pooling the four populations, but the reason is that the exposure ranges did vary quite substantially between the different populations. Undoubtedly, it would have been valuable to have a more extensive exposure profile of the POPs than the two biomarkers analysed in this project, and we plan to perform such analyses on a stratified sub-sample from the study populations. On the other hand, it should be kept in mind that for practical and economical reasons it would have been impossible to analyse more than 3,000 samples, as in the present project, for a more detailed POP exposure profile. When performing epidemiological studies on reproductive outcomes, large enough sample sizes are needed to ensure a reasonable statistical power. Thus, a trade off is needed between costs for obtaining large enough number of samples and costs for detailed analyses of each sample, in order to obtain the most efficient study design.

The analysis of the relative impact of different determinants for variation in serum concentrations of the POP biomarkers showed a consistent association with *gender*. For the four geographical regions the median CB-153 concentrations were 55–126 % higher among the men. The corresponding figures for p,p'-DDE were 39–87 %. The results are in concordance with previous studies of Inuits [19], frequent consumers of Great Lakes sport fish [20], and Swedish fishermen's families [7,8]. The main reason for the observed gender difference is probably excretion of POP by breast feeding.

Increasing *age *was also a most consistent determinant of higher serum concentrations of CB-153 and p,p'-DDE in both men and women. This is in line with earlier findings [20-23], and could be caused by both an age-dependent bioaccumulation of the persistent and lipophilic compounds and higher exposure concentrations in the past detectable in the more elderly; a birth cohort effect [24]. Previous Swedish data on women support that the birth cohort effect is of great importance [22]. This is due to that the highest POP contaminant concentrations in different food items were found in the 1970's and the concentrations have continuously decreased since then. The effect of this is that for a given calendar year of birth, CB-153 in plasma decreased with age, from the time point when steady state of body burden was reached for this compound with a biological half-live of many years, and onwards. Thus, in a longitudinal study design, the plasma concentration of CB-153 will decrease with age, from time of steady state, while applying a cross-sectional approach, older subjects will have higher concentrations than younger ones.

When evaluating the impact of age on serum concentrations of POP in women, one has to keep in mind that age is associated with *parity a*nd therefore also with *total length of breast-feeding*, which works against an observed increase in serum concentrations of POP with age. Consequently, there was in the present study an independent negative association between life-long total length of breast-feeding and POP concentrations in serum among the Swedish women. In the multivariate models breast-feeding increased the explained variance for the POP biomarkers among the Swedish women with, as most, 6 %. Negative associations between total length of breast-feeding and PCB concentrations in mother's milk [25-27], as well as blood plasma [22] have previously been shown. About a third of the Inuit women were primipara, and parity did only increase the explained variance in the multivariate models with 1–2 %. The exposure determinant *seafood *varied between the populations. Among Inuits seafood comprised both sea mammals and fish, whereas for Sweden it comprised locally caught fish from the Baltic Sea (east coast) and the North Sea (west coast), respectively. For both male and female Inuits there were clear positive association between consumption of seafood and the POP markers. Among the fishermen's wives from Sweden the area of living modified the effect of fish consumption on POP concentrations. The impact of seafood (per weekly meal) was greater among women from the Swedish east coast, which is explained by the higher concentration of POP contamination in fatty fish from the Baltic Sea as compared with fish from the North Sea at the west coast [28]. In a previous Swedish study on elderly women from the general population [29] and in US studies [24], fish consumption was associated with exposure to PCBs but not to p,p'-DDE. A positive association between fish consumption and PCB was observed for both genders among high consumers of sport fish from the Great Lakes [20]. The seafood consumed in the Warsaw and Kharkiv populations was mainly fish from freshwater lakes and this did not seem to be a substantial exposure source for CB-153 or p,p'-DDE.

There was in the present study no consistent association between *BMI *and serum concentrations of CB-153 or p,p'-DDE. The positive association between BMI and p,p'-DDE among the Swedish fishermen was the only significant finding, but the explained variance in the multivariate model did only increase with 1 % by including BMI in the model. In a previous study POPs were negatively associated with BMI among young and middle-aged male Inuits from Greenland [14], but we did not observe such an association. The previous literature gives no clear picture of the association between BMI and body burdens of POP. BMI was a predictor of serum p,p'-DDE in some studies [21,30,31], but not in other [24,32]. Two studies on women from the US found a negative association between BMI and serum concentration of higher chlorinated or total PCB [23,31], whereas no association was found between total PCB concentration and BMI in two other studies [21,24]. A positive association between BMI and PCB was observed for both genders among high consumers of sport fish from the Great Lakes [20]. The association with BMI varied between congeners in a Swedish study of elderly women [29]. For some PCB-congeners (CB-105 and CB-118) and p,p'-DDE positive associations were seen, while negative associations were observed for some other PCB congeners (CB-156 and CB-180), and for e.g. CB-153 no significant association at all was observed. Thus, there may be compound-specific differences in the modulating effects of BMI on serum POP concentrations. Moreover, the inconsistent results with respect to the association between BMI and POP in serum may be explained by the timing of blood sampling in relation to when the period of more substantial dietary POP exposure had taken place [33]. If this exposure had been recent, subjects with high BMI, i.e. with large adipose distribution volumes, should be expected to have negative association between BMI and lipid adjusted POP concentrations in serum. On the other hand, if the blood samples were drawn many years after end of a more substantial exposure than the present one, and the subjects were old enough to have reached a steady state of their body burdens of POPs, considering the long biological half-lives of many of these compounds, you could expect to find a positive association between BMI and POP in serum. This reasoning fits with our finding of a positive association between BMI and p,p'-DDE among the middle-aged and elderly Swedish fishermen, who decades ago had been substantially more exposed to POPs through consumption of fatty fish from the Baltic Sea [28].

Among the Inuit women we observed positive associations between current *smoking *and serum concentrations of both CB-153 and p,p'-DDE. However, by including smoking in the multivariate model the explained variance did only increase with 1 %. In contrast, a negative association between current smoking and p,p'-DDE was observed among the women from Warsaw, whereas no association between POP markers and smoking was observed among women from Kharkiv. In Inuit men and men from Kharkiv, smoking was not associated with any of the POP biomarkers, whereas there was a negative association between smoking and CB-153 among Swedish fishermen. Thus, is it possible to explain this ambiguous pattern of associations? Our results are in accordance with previous studies of Inuit women from Greenland [14,34], and also of Inuit women from Canada [35]. It is, however, of interest that no such association was found among the Caucasian control women in the latter study. Moreover, in two Swedish studies on elderly women and fishermen's wives, respectively, no association between POP and smoking was seen [22,29]. In contrast to the present results, previous studies on male Inuits from Greenland showed in multivariate analyses positive associations between smoking and PCB in serum [37]. It has been proposed that smoking can affect uptake or metabolism of POPs, e.g. by influencing the CYP-450 enzyme system [14], but this seems to be an unlikely explanation as induced CYP-450 system (by smoking or PCB) would rather result in lower PCB concentrations due to increased formation of hydroxylated metabolites. It has been reported an association between consumption of sea mammal fat and tobacco smoking among Canadian Inuit [15], and a more probable explanation for the presently observed weak association between smoking and POP in Inuit women might be a residual confounding of diet.

The participation rate varied considerably between the different populations (10–87 %). We have, however, no reason to suspect that the subject's decision to participate was affected by any knowledge of their individual POP exposure. It was not possible to adjust for participation rate as this is a population characteristic and not an individual characteristic. Concerning the external validity of the present study, our conclusions are restricted to the age groups studied. Thus, we cannot say anything about serum concentrations of POP biomarkers in children, adolescents or in women past their reproductive age (except for Sweden). Three of the female study populations comprised pregnant women, which could hamper a comparison with non-pregnant women of the same age. However, gestational length affected the POP biomarker concentrations only in the Kharkiv population. Adjusting for the other determinants, each extra week of pregnancy in Kharkiv women was associated with a decrease of the POP biomarkers with 1 %. We think that the participating men from Greenland, Warsaw and Kharkiv were representative for the general population in the same geographical area, whereas the Swedish fishermen were more highly exposed to POPs than the general Swedish population.

We used CB-153 and p,p'-DDE in serum as index biomarkers of exposure to POPs and there are several previous studies supporting this approach [12-15]. It must, however, be taken into consideration that the relative concentrations of non-coplanar and co-planar PCBs can differ between regions depending on varying exposure sources. Studies of Inuit populations from Canada support in general the use of CB-153 as a surrogate marker of exposure to non-dioxin like PCBs present in the Arctic food-chain [15]. However, the ratio between serum concentrations of CB-153 (and other non-coplanar congeners) and co-planar PCBs was higher in Canadian Inuits than in Canadian Caucasians from the Arctic area [37], which calls for caution using CB-153 as a global exposure marker for POP.

POP concentrations in blood have previously been analyzed in several studies both from Greenland and Sweden. Samples drawn in 1994–1997 from pregnant women from the Disco Bay area at the west coast of Greenland showed that both CB-153 and p,p'-DDE were at that time found in about 50 % higher concentrations than in the present study [7,38,39]. Glynn et al. monitored in 1996–1997 CB-153 and p,p'-DDE in serum from elderly women (54–75 years) living on the Swedish east coast or around the Swedish large lakes [29]. The concentrations were about two times that of the Swedish fishermen's wives in the present study. These results are in accordance with other studies also showing a trend of decreasing POP exposure during the last decades [40-47]. Asplund et al. monitored in 1987 CB-153 and p,p'-DDE in plasma from Swedish men (25–56 years) with low to high consumption of fatty fish from the Baltic Sea [48]. The Swedish low consumers in 1987 had POP concentrations twice as high as the Swedish fishermen in the present study. A similar study, with samples collected in 1991, showed among men with low consumption of fatty fish from the Baltic Sea, CB-153 and p,p'-DDE in the same range as those presently reported for Swedish fishermen, while the concentrations in the high consumer group was more than twice as high [49]. Thus, these results indicate that there has been a decrease in POP concentrations in blood in both Greenland and Sweden during the last decade(s). For Warsaw and Ukraine there are no reports of CB-153 and p,p'-DDE in blood that can be used for time trend analyses. However, analysis of maternal milk from Ukraine, donated 1993–1994, showed a median p,p'-DDE concentration of 2457 ng/g lipid [11], which is considerably higher than the median lipid adjusted p,p'-DDE concentration in serum of 650 ng/g lipid in the present study, indicating a rather dramatic decrease in exposure during the last decade.

## Conclusion

Serum concentrations of CB-153 were much higher in the Inuit and Swedish fishermen's populations as compared with the populations from Eastern Europe, whereas the exposure pattern was different for p,p'-DDE showing highest concentrations in the Kharkiv population and relatively low concentrations in the Swedish fishermen's populations.

The degree of correlation between the two POP biomarkers varied considerably for the different populations, which underlines that the exposure sources obviously differ between countries and that the choice of representative biomarkers of overall POP exposure has to be based on an analysis of the specific exposure situation for each population.

Age and gender affected the serum POP concentrations in all populations whereas the associations with BMI and smoking were inconsistent. Dietary seafood and area of residence were of importance for the POP biomarker concentrations only in the Inuit and Swedish populations.

## List of Abbreviations

BMI body mass index

CB-153 2,2',4,4',5,5'-hexachlorobiphenyl

CV coefficient of variation

p,p'-DDE 1,1-dichloro-2,2-bis (*p*-chlorophenyl)-ethylene

DDT dichlorodiphenyl trichloroethane

PCBs polychlorinated biphenyls

POPs persistent organochlorine pollutants

TTP time to pregnancy

## Competing interests

The author(s) declare that they have no competing interest.

## Authors' contributions

JPB, AG and LH initiated the project. JPB was main responsible for raising funding for the project. ARH, GT, HSP, JKL, KG, and VZ have been responsible for collecting all blood samples and for obtaining the interview data. BAGJ and CL were responsible for the chemical analyses of the POP biomarkers. GT had main responsibility for creating the joint database. LR performed the statistical analyses. LH, BAGJ, LR, JPB, GT and ARH helped to draft the manuscript. All authors participated in the design of the study and have read and approved the final manuscript.
